# Supplementary material for: Nuclear-driven production of renewable fuel additives from waste organics
Source: Commun Chem. 2021 Sep 17;4:132. doi: 10.1038/s42004-021-00572-5 (PMC9814337; doi:10.1038/s42004-021-00572-5)
Supplement: Supplementary file 1 — Supplementary Information [file 42004_2021_572_MOESM1_ESM.pdf]

Supplementary Information

Nuclear-driven production of renewable fuel additives from waste organics

Arran George Plant[1], Bor Kos[2], Anže Jazbec[2], Luka Snoj[2], Vesna Najdanovic-Visak[3*] & Malcolm John Joyce[1*]

[1]Engineering Department, Lancaster University, Lancaster, UK.
[2]Jožef Stefan Institute, Ljubljana, Slovenia.
[3]Chemical Engineering and Applied Chemistry (CEAC), Energy & Bioproducts Research Institute (EBRI), Aston University, Birmingham, UK

* email: v.najdanovic@aston.ac.uk; m.joyce@lancaster.ac.uk

Supplementary Table 1: Mixed-field neutron+γ-ray or only γ-ray radiolysis of glycerol: qualitative products detected using liquid sampling GC-MS techniques. Displayed products are for large absorbed doses (>20 kGy). Molecule similarity comparison against the NIST11 MS spectrometry database, products were confirmed with analytical standards where applicable.

**Number**	**Product**	**Peak Retention Time (min.)**	**% Similarity**	**Confirmed Using Standards (Y/N)**	**Quoted in Literature of Glycerol or Similar Alcohols (Y/N)?**
1	Formaldehyde	1.7	95	N	Y[1]
2	Acetaldehyde	2.1	96	Y	Y[1]
3	Methanol	2.2	90	Y	Y[1]
4	Acetone	3.6	97	Y	Y[2,3]
5	Glycoaldehyde Dimer	6.2	97	Y	Y[2]
6	Acetol (Hydroxyacetone)	11.0	98	Y	Y[2]
7	Glyceraldehyde	14.9	92	N	Y[2]
8	Solketal ((2,2-Dimethyl-1,3-dioxolan-4-yl)methanol)	15.4	98	Y	N
9-23	14 Unidentifiable Products*		<88	-	-

***Radiolysis products yet unidentified – The majority are secondary and higher-order products with longer retention times.

Supplementary Table 2: Dose dependence of concentrations and radiation chemical yields (*G*-values) of acetol and solketal from irradiated neat glycerol samples (Figures 1 a,b).

	Dose Type (reactor mode)	only γ rays (Shutdown)						Control	Mixed-field neutron+γ rays (Operational)						
	**Absorbed Dose kGy**	20	40	50	60	80.2	100	NA	20	40	50.4	60	80.2	100
**Acetol**	**Concentration in neat Irradiated Sample (μg ml^{-1})**	1083	1902	2955	2944	4161	4362	<LOD	257	597	963	899	1681	3883
	***G*-value (μmol J^{-1})**	0.58	0.51	0.63	0.53	0.56	0.47	<LOD	0.14	0.16	0.20	0.16	0.22	0.42
**Solketal**	**Concentration in Irradiated Sample (μg ml^{-1})**	62	175	379	410	733	798	<LOD	<LOQ	<LOQ	62	76	228	716
	***G*-value (μmol J^{-1})**	0.019	0.026	0.045	0.041	0.055	0.048	<LOD	<LOQ	<LOQ	0.0074	0.0076	0.0171	0.0430

*LOQ = Limit of quantitation

*LOD = Limit of detection

Supplementary Table 3: Linear trend analysis values for the absorbed dose-*G* value relationship of acetol and solketal from neat glycerol samples. (Shown in Figures 1a and b). Linear trends to the equation; $y=mx+c$, SD=Standard Deviation, χ^2_ν = Chi squared, ν =degrees of freedom, n=number of data points in dataset.

Product	Irradiation Type	*m* (μmol J^{-1} kGy^{-1})	*c* (μmol J^{-1})	SD	χ^2_ν (ν,n)	P (0<X^2<χ^2_ν)
**Acetol**	**Only γ rays**	-1.1×10^{-3}	0.604	0.057	3.1 (4, 6)	0.47
	**Neutron+γ rays**	2.2×10^{-3}	0.077	0.037	12.8 (4, 6)	0.99
**Solketal**	**Only γ only**	5.23×10^{-4}	0.0083	0.0045	8.4 (4,6)	0.92
	**Neutron+γ rays**	6.83×10^{-4}	– 0.0305	0.0145	3.4 (2,4)	0.98

Supplementary Table 4: Dose rate dependence of concentrations and *G*-values of acetol and solketal using 50 kGy mixed-field neutron+γ-rays irradiation (Figures 1c, d).

	**Dose Rate (kGy/min)**	0.52	1.31	3.27	8.17
**Acetol**	**Concentration in Neat Irradiated Sample (µg ml^{-1})**	1185	1109	1229	794
	***G*-value (µmol J^{-1})**	0.25	0.24	0.26	0.17
**Solketal**	**Concentration in Neat Irradiated Sample (µg ml^{-1})**	117	57	16	24
	***G*-value (µmol J^{-1})**	0.0141	0.0069	0.0019	0.0029

Supplementary Table 5: Concentrations, *G*-values and % glycerol conversion values of acetol and solketal from all 50 kGy irradiated mixtures and unirradiated control mixtures (Figure 2a, b, c, d).

	**Dose Type (reactor mode)**	**only γ rays (Shutdown)**							**Mixed-field neutron+γ rays (Operational)**							**Control (Unirradiated)**		
	**Mixture Type**	Neat	Gly+H_2O			Gly+Acet.+H_2O			Neat	Gly+H_2O			Gly+Acet.+H_2O			Gly+Acet.+H_2O		
	**Glycerol Mol%**	100	31	27	16	20	13	11	100	31	27	16	20	13	11	20	13	11
**Acetol**	**Concentration in Irradiated Sample (µg ml^{-1})**	2955	8002	5670	3808	2387	1765	1426	963	2075	1381	1138	479	331	223	<LOD	<LOD	<LOD
	***G*-value (µmol J^{-1})**	0.63	1.83	1.35	0.92	0.63	0.49	0.42	0.20	0.48	0.33	0.27	0.13	0.09	0.07	NA	NA	NA
	**% Molar Yield from Glycerol**	0.29	1.22	0.99	0.85	0.63	0.66	0.73	0.01	0.32	0.24	0.18	0.13	0.13	0.11	<LOD	<LOD	<LOD
**Solketal**	**Concentration in Irradiated Sample (µg ml^{-1})**	379	144	90	65	10279	7190	4917	62	<LOQ	<LOQ	<LOQ	1181	801	600	850	503	288
	***G*-value (µmol J^{-1})**	0.045	0.019	0.012	0.01	1.53	1.11	0.80	0.01	<LOQ	<LOQ	<LOQ	0.18	0.12	0.09	NA	NA	NA
	**% Molar Yield from Glycerol**	0.02	0.01	0.01	0.01	1.53	1.52	1.41	0.00	<LOQ	<LOQ	<LOQ	0.13	0.13	0.11	0.13	0.11	0.08

*LOQ = Limit of quantitation

*LOD = Limit of detection

Supplementary Table 6: Acetic acid concentrations, *G*-values and acetone % conversion from aqueous glycerol-acetone mixtures with 50 kGy of absorbed doses.

	Dose Type (reactor mode)	only γ rays (Shutdown)			Mixed-field neutron+γ rays (Operational)		
	**Acetone Mol %**	28	34	48	28	34	48
**Acetic Acid**	**Concentration in Irradiated Sample ($\mu g\ ml^{-1}$)**	233	431	486	232	235	259
	***G*-value ($\mu mol\ J^{-1}$)**	0.076	0.15	0.17	0.076	0.08	0.093
	**% Molar Yield from Acetone**	0.079	0.126	0.011	0.079	0.069	0.056

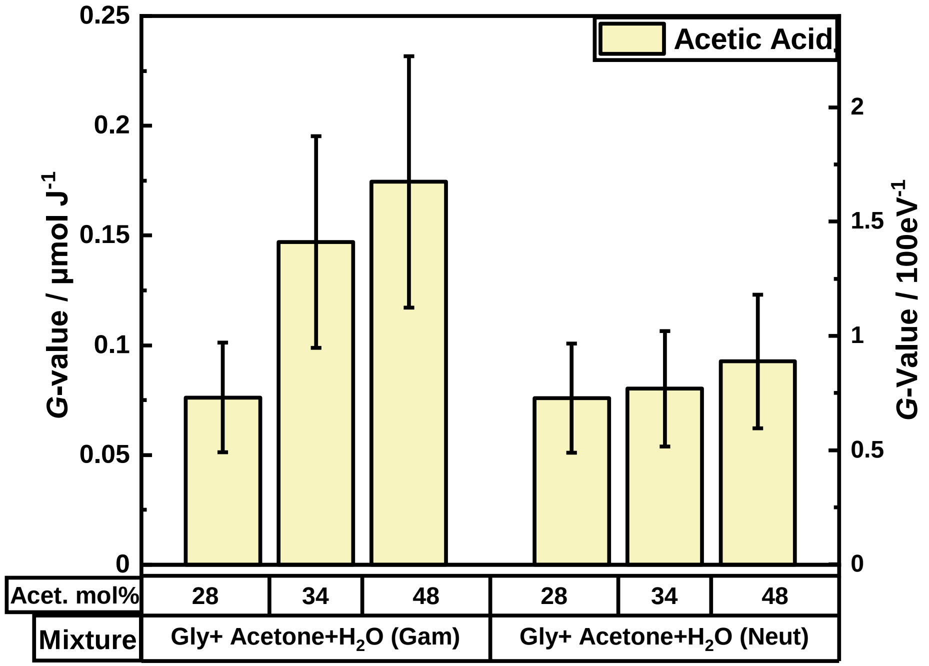

Supplementary Figure 1: *G*-values of acetic acid from the ternary (glycerol + acetone + water) mixtures using only γ-ray and neutron+γ-ray irradiations. Samples have been either irradiated with 50 kGy γ ray only irradiations with an average dose rate of 40 Gy min^{-1} or 50 kGy neutron+γ-ray mixed field irradiations with dose rates of 3260 Gy min^{-1}**.** Error bars represent the calculated instrumental errors for each sample.

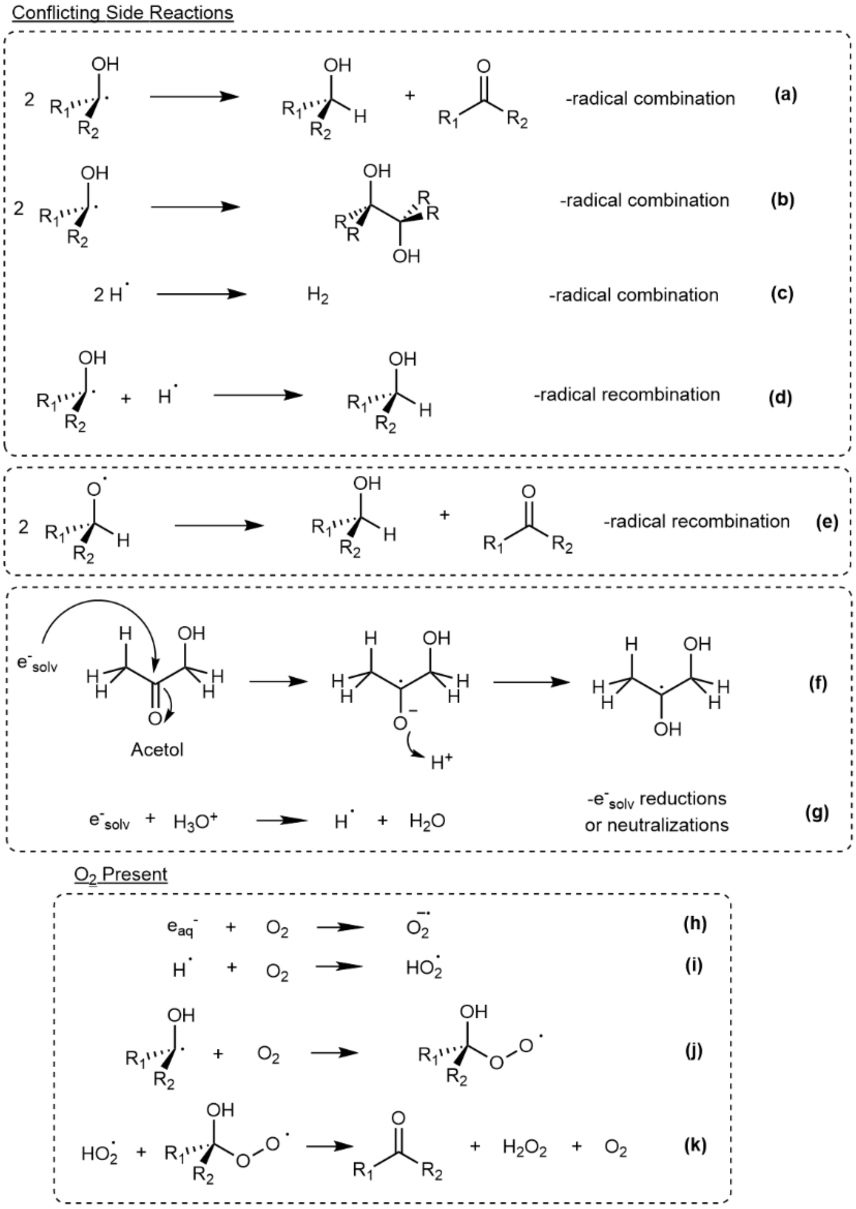

Supplementary Figure 2: Conflicting reactions affecting acetol: a, to **e**, are interspur radical combinations reactions promoted by the overlapping of spurs with high LET and high dose-rates. **f**, reduction of acetol via e_{solv}^{-}. **g**, neutralization of the hydronium ion. **h**, superoxide anion formation. **i**, hydroperoxy ($HO_2^{\bullet -}$) radical formation. **j**, Peroxyl radical ($RO_2^{\bullet}$) formation. **k**, radical combination of peroxyl radicals for aldehyde formation. (R_1 and R_2 = -CH_2OH for α-carbon or H and –CHOH-CH_2OH for β-carbon radicals, respectively.)

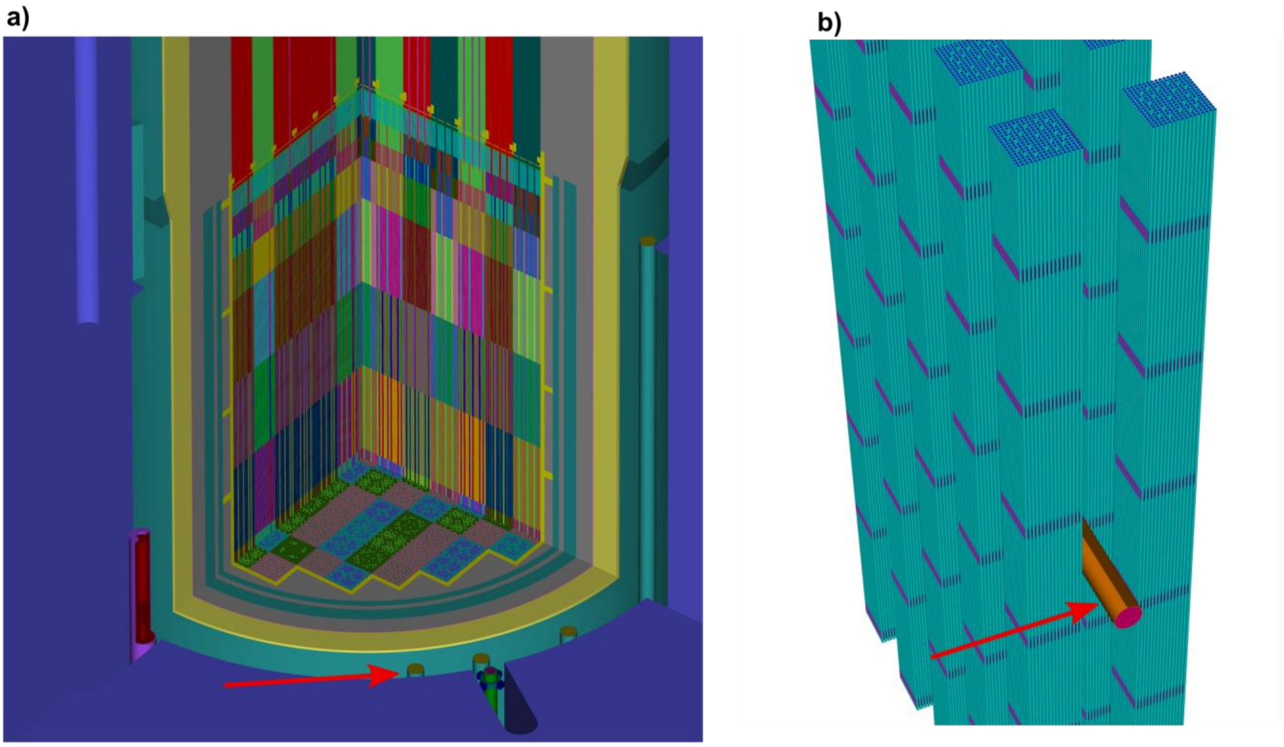

Supplementary Figure 3: MCNP models for scale-up calculations. a, 2 GWth PWR core and **b,** a PWR spent fuel pool with 10 fuel elements extrapolated to 1780 total elements for capacity calculations. Red arrows indicate the irradiation positions for organic mixtures in each scenario.

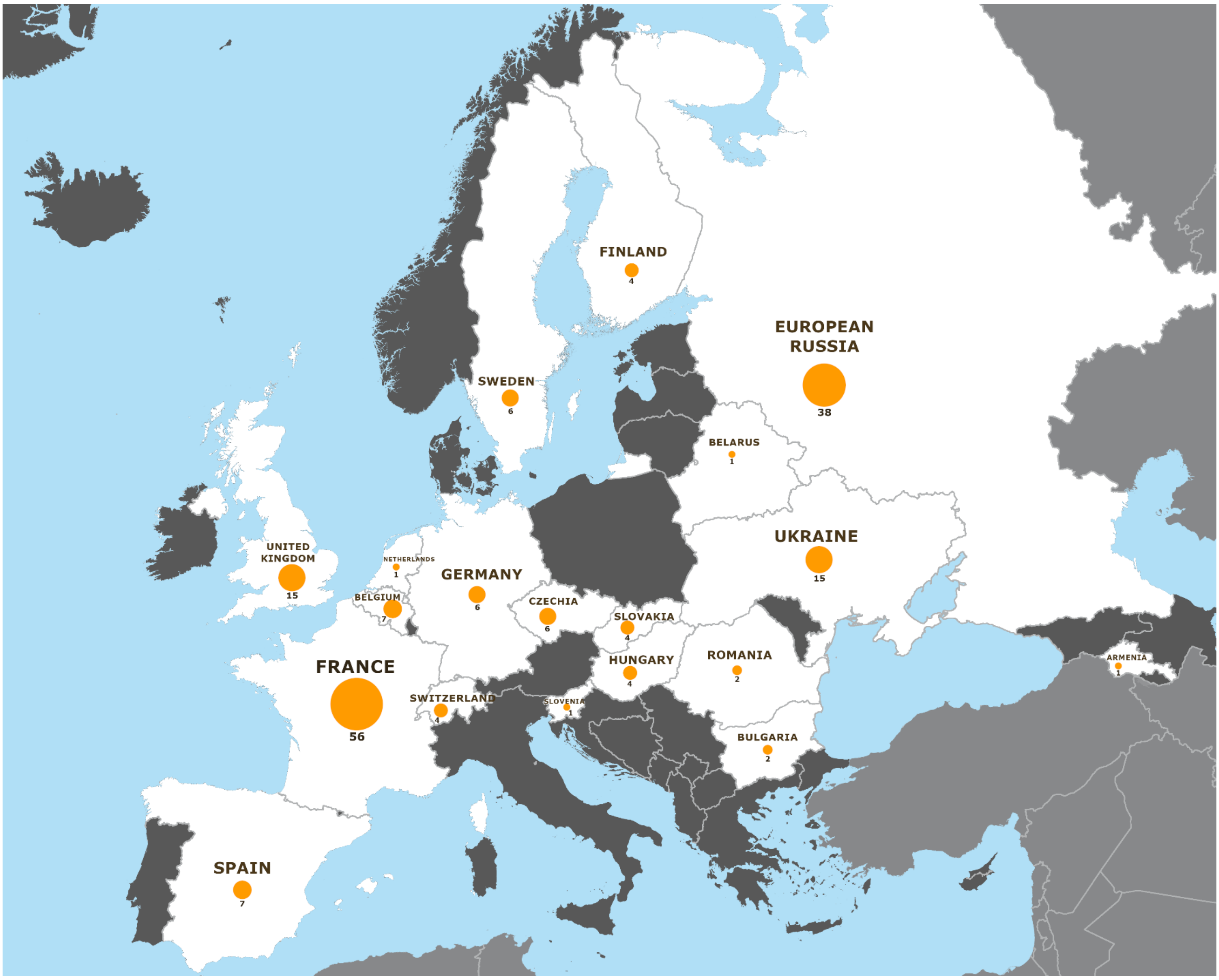

Supplementary Figure 4: Geographical map of Europe displaying the location of 180 operational reactors by country as of 11/03/2021. Areas of orange circles indicate the number of reactors within that country. It is assumed that the number of reactors is indicative of the spent fuel capacity available for co-production for each country. Base Map © Maproom.

Supplementary References

1. Freeman, G. R. *Radiation Chemistry of Ethanol: A Review of Data on Yields, Reaction Rate Parameters, and Spectral Properties of Transients. (National Bureau of Standards, 1974)*
2. Baugh, P. J., Moore, J. S., Norris, A. F., & von Sonntag, C. *γ-Radiolysis of N2O-saturated aqueous glycerol solutions: Product yields and free radical mechanism*. *Radiation Physics and Chemistry (1977)* 20.3 (1982): 215-222.
3. Spinks, J. W. T. & Woods, R. J. in *An introduction to radiation chemistry* (1990).
